# Compound Danshen Dripping Pill for Treating Early Diabetic Retinopathy: A Randomized, Double-Dummy, Double-Blind Study

**DOI:** 10.1155/2015/539185

**Published:** 2015-09-20

**Authors:** Dan Luo, Yali Qin, Wei Yuan, Hui Deng, Youhua Zhang, Ming Jin

**Affiliations:** ^1^Department of Graduate School, Beijing University of Chinese Medicine, No. 11, North 3rd Ring East Road, Chaoyang District, Beijing 100029, China; ^2^Department of Ophthalmology, China-Japan Friendship Hospital, Yinghua Donglu, Chaoyang District, Beijing 100029, China

## Abstract

This randomized, double-dummy, double-blind study was to observe the therapeutic effects of compound Danshen dripping pill (CDDP) in treating early diabetic retinopathy (DR). All the 57 type 2 diabetes cases in nonproliferative diabetic retinopathy (NPDR) stage were divided into two groups randomly: 28 cases treated with CDDP as the treated group and 29 cases treated with calcium dobesilate as the control group. The best corrected visual acuity (BCVA) in the treated group was significantly improved after treatment when compared to that before treatment (*P* < 0.05). Mean defect (MD) of visual field, hemorrhage area of the fundus, microaneurysm number, fluorescent leakage area, and capillary nonperfusion area evaluated by visual field, fundus photography, and fundus fluorescein angiography in the treated group had the same results as BCVA. However, there was no statistical difference in each index between the two groups. No obvious adverse events with clinical significance occurred. Our present study showed that CDDP has a similar improvement and safety to calcium dobesilate for NPDR. In future DR treatments, CDDP may function as the auxiliary drug.

## 1. Introduction

Diabetic retinopathy (DR) is a common microvascular complication of diabetes. DR is characterized by microaneurysm, exudation, neovascularization, vitreous hemorrhage, and so on in the retinal microvasculature, which can cause different degrees of vision loss or irreversible blindness. Approximately, more than 30% of these patients have DR [1]. Moreover, one-quarter of DR patients develop severe visual impairment [2], which is mainly attributed to diabetic macular edema and proliferative diabetic retinopathy (PDR) [3]. Long-term high blood sugar or blood glucose fluctuations can cause the structure of blood vessels, blood composition, hemodynamic abnormalities, and a series of metabolic disorders within the body [4]. At present, long-term control of blood sugar, blood pressure, and blood lipid levels in the normal range is still an important measure to delay development of DR. For nonproliferative diabetic retinopathy (NPDR), laser photocoagulation [5] and surgical treatment [6] are the most commonly used therapeutic methods, but, for pathological changes with the retina or the macular hemorrhage and edema, antivascular endothelial growth factor (VEGF) drugs are used [7] in clinical treatment. However, these therapeutic methods cannot reverse the existing retinal damage. Moreover, some adverse events are associated with these treatments [8]. Visual acuity could be severely damaged if NPDR is not successfully prevented and cured. Although DR has been widely studied, no effective and well-recognized drugs can prevent or reverse the progress of DR. Therefore, a new therapeutic strategy should be developed to alleviate DR progression at an early stage. Chinese herbal medicines are alternative therapeutics that have been used for thousands of years in China. These medicines are now acceptable in the world. Hence, these medicines should be explored and used for treating DR.

Traditional Chinese medicine (TCM) considered that DR is developed because of blood stasis, which causes hemorrhage and angiogenesis [9]. In Western medicine, vascular leakage caused by microangiopathy leads to diabetic macular edema and capillary occlusion, followed by retinal ischemia and increased levels of VEGF, which are responsible for DR development from NPDR to PDR [3]. Therefore, the improvement of ischemia and the protection of normal microvasculature in retinal tissue save the visual acuity of patients with diabetes and inhibit DR progression.

Several Chinese herbs can remove blood stasis including Danshen,* Salvia miltiorrhiza*. A well-established formulation of* S. miltiorrhiza* called compound Danshen dripping pill (CDDP) as the first new drug through the American FDAII clinical trials has been used to treat cardiovascular diseases [10], which is still in trial stages in DR treatment. Lian et al. [11] reported that, compared with the placebo, CDDP has definite efficacy and safety for treating NPDR. To compare and detect the efficacy of CDPP in treating DR further, we performed a randomized, double-dummy, double-blind study. Calcium dobesilate was chosen to be the control agent. The efficacy and safety of calcium dobesilate as a vasoprotective agent have been confirmed in many randomized clinical controlled trials because of its antioxidant and antiapoptotic properties, which can improve diabetic endothelial dysfunction and slow vascular cell proliferation [12] to effectively treat DR at the systematic and local ocular levels [13].

## 2. Methods

### 2.1. Clinical Trial Design

From June 2010 to April 2013, 60 patients diagnosed with NPDR because of type 2 diabetes were recruited in the Department of Ophthalmology, China-Japan Friendship Hospital. The Institutional Review Boards and Ethics Committees of the China-Japan Friendship Hospital in accordance with the provisions of the Declaration of Helsinki approved the study protocol. All patients signed the consent form. The inclusion criteria were as follows: (1) HbA1C blood was below 8% in the past 3 months and (2) the fundus condition had no significant aggravating period. The exclusion criteria were as follows: (1) patients with other retinopathy diseases, glaucoma, or influencing fundus examination diseases; (2) patients with uveitis, retinal detachment, or optic nerve diseases; (3) patients with severe heart, liver, or hematopoietic system diseases; (4) patients with renal failure caused by diabetic nephropathy; (5) pregnant or lactating patients or those with psychosis; and (6) patients participating in other clinical trials or taking other drugs that affect the results of this study.

CDDP was prepared by Tasly Pharmaceutical Group Co., Ltd. (Tianjin, China), which includes Danshen (*S. miltiorrhiza*), notoginseng (*Panax notoginseng*), and borneol. Patients in CDDP group were orally administered 15 CDDP pills at a time, thrice a day, whereas patients in the calcium dobesilate group were orally administered 500 mg calcium dobesilate (Xi'an Lijunsha Pharmaceutical Co., Ltd., China) at a time, thrice a day. One CDDP pill contains 27 mg of herbal medicine. The treatment period was 3 months.

### 2.2. Evaluation of Effectiveness

The primary outcome was the best corrected visual acuity (BCVA). Visual acuities were converted to logarithm of the minimum angle of resolution (Log MAR) values for analysis. The criteria of visual acuity were as follows: improvement ≥ 4 lines indicated excellent effective rate; improvement of 2 to 3 lines indicated effective rate; improvement of 1 line or no changes indicated stable rate; and decline ≥ 2 lines indicated ineffective rate.

The secondary outcomes included mean defect (MD) of visual field, hemorrhage area of fundus, the microaneurysm number, fluorescent leakage area, and capillary nonperfusion area. MD of the visual fields of all eyes was tested using the Octopus 101 perimeter (InterZeag Inc., Switzerland) under the aid of an examiner. Each patient should complete the perimetry at least twice to judge the repeatability of the result. We required false positive or negative rates < 30% or reliable factor < 15%. The MD of the visual field was evaluated over the normal range, which was ≥ 10%, indicating excellent effective rate; ≥0 and <10% indicated effective rate; and <0 indicated ineffective rate.

After mydriasis, the patients were given 3 mL of 20% sodium fluorescein through elbow vein injection for the fundus fluorescein angiography (FFA; Topcon TRC-50DX, Topcon Corp., Tokyo, Japan) image monitoring for 20 min. The microaneurysm number, hemorrhage area of fundus, fluorescent leakage area, and capillary nonperfusion area were evaluated in the four quadrants of the fundus with FFA. The criteria of the microaneurysm number were as follows: reduction of ≥10% indicated excellent effective rate; reduction of ≥0 and <10% indicated effective rate; and increased value indicated ineffective rate. The extent of the area was estimated using optic disc area as the basic unit. The criteria of decreasing rate of the area were as follows: ≥ 10% had excellent effective rate; ≥0 and <10% had effective rate; and increased value had ineffective rate.

### 2.3. Safety Evaluation and Adverse Events

Before and after treatment, the blood routine, liver and kidney function, fasting blood sugar, and urine sugar tests were performed for safety evaluation. During the study, any adverse events related to CDDP were recorded, including clinical signs and symptoms.

### 2.4. Statistical Analyses

All data of the results were presented as the means ± standard deviations and analyzed with SPSS 17.0 (SPSS Inc., Chicago, IL, USA). The basic characteristics were determined by descriptive analysis. Paired* t*-test was used to analyze the before- and after-treatment comparison of each group. Two-sample mean comparison was conducted by independent samples *t*-test. Disaggregated data correlation was conducted by Chi-square test. Differences with *P* < 0.05 were considered statistically significant.

## 3. Results

### 3.1. Baseline Characteristics

After eliminating 3 cases for angle-closure glaucoma and cataract, a total of 57 eligible cases (114 eyes) were enrolled into this study. The statistical analysis was conducted by dividing the samples into two groups by random allocation. As shown in Table 1, in the treated group, 18 patients (64.3%) were male, and 10 patients (35.7%) were female. The mean ± standard deviation age was 59.54 ± 7.46 years (range: 43–67 years old), and the duration was 14.52 ± 3.29 years (range: 7–21 years old). In the control group, 19 patients (65.5%) were male, and 10 patients (34.5%) were female. The mean ± standard deviation age was 57.86 ± 10.03 years (range: 45–64 years old), and the duration was 15.74 ± 3.63 years (range: 9 to 21 years old). According to the International Clinical Classification Systems for DR [14] in 2002, the numbers of patients with mild to severe NPDR were 18, 22, and 16 in the treated group and 19, 25, and 14 in the control group. No significant differences were observed between both groups on age, gender, duration of disease, and stage of disease (*P* > 0.05). Hence, the patient characteristics in both groups were similar.

### 3.2. Changes in BCVA

The BCVA changes of both groups before and after the treatment are listed in Table 2. After the treatment, the BCVA of both groups significantly improved compared with the BCVA before the treatment (*P* < 0.05). The BCVA of the treated group was 0.14 ± 0.17 (Snellen equivalent, 20/28), and that of the control group was 0.17 ± 0.18 (Snellen equivalent, 20/30). However, no significant difference was observed between the two groups before and after the treatment (*P* > 0.05). No significant difference was observed between the two groups in “improvement ≥ 4 lines,” “2 to 3 lines,” “1 line or no changes,” and “decline ≥ 2 lines” for BCVA evaluation after the treatment (*P* > 0.05). According to the criteria of visual acuity, the total effective rates of the treated and control groups were 71.43% and 62.07%, respectively. Comparatively, the former was more effective than the latter.

### 3.3. Changes in Visual Field

**Figure 1:**
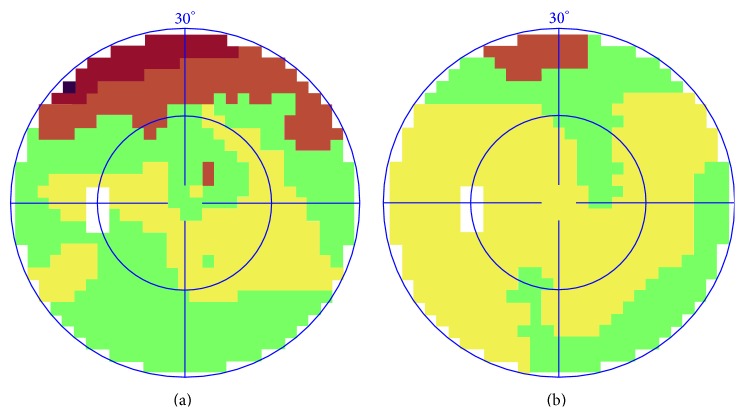
Representative images of a case before and after CDDP treatment. (a) Visual field before treatment. (b) Visual field after treatment.

Visual field means the space scope felt by the patient with single eye looking in the due front direction. The defect of visual field showed the lesions in the relevant fundus parts. Colors are used to indicate the degree: yellow means normal, and green, red, and black indicated that the lesions were worsened (Figure 1). The changes of the MD of the visual field before and after the treatment are shown in Table 3. Before the treatment, MD of the treated group was 4.61 ± 3.54. Compared with the control group (5.08 ± 4.58), no remarkable difference was observed (*P* > 0.05). MD value of the visual field notably decreased compared with before in the treated group, and the differences were remarkable (*P* < 0.05). The MD value of the visual field notably decreased compared with before in the control group (*P* < 0.05). However, no remarkable differences were observed between the groups after treatment (*P* > 0.05). According to the criteria of MD, the total effective rate of the treated group was 62.50%, and that of the control group was 67.24%. No significant difference was observed between the two groups in “≥10%,” “≥0 and <10%,” and “<0” for MD evaluation after treatment (*P* > 0.05). One example of visual field changes with NPDR was shown in Figure 1.

### 3.4. Changes in Microaneurysm Number

**Figure 2:**
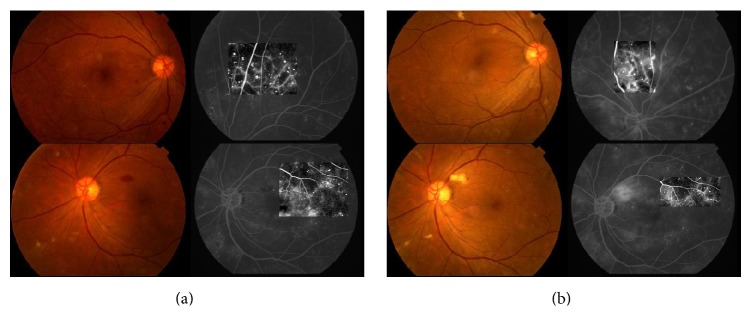
Representative images of a case before and after CDDP treatment. (a) Color fundus photograph and FFA photograph before treatment. (b) Color fundus photograph and FFA photograph after treatment.

Microaneurysm number can reflect long-term progression in DR [15]. Significant changes were found after treatment as shown in Table 4 and Figure 2. The microaneurysm number decreased in each group compared with that before treatment (*P* < 0.05), but no significant differences were found between the groups after treatment (*P* > 0.05). After treatment, the small blood spots disappeared, and the effect was significant. Table 4 shows that both CDDP and calcium dobesilate had effects in treating microaneurysm and small blood spots. The total effective rates of the treated and control groups were 75.00% and 77.59%, respectively. No significant difference was observed between the two groups in “≥10%,” “≥0 and <10%,” and “<0” for microaneurysm number evaluation after treatment (*P* > 0.05).

### 3.5. Changes in the Hemorrhage Area of the Fundus

Changes in hemorrhage area of fundus are shown in Table 5 and Figure 2. The significant difference was found in the treated group after the treatment compared with that before treatment on the hemorrhage area (*P* < 0.05). The control group had a similar result. Meantime, compared with the treated group, the control group did not have a noticeable advantage in hemorrhage reduction (*P* > 0.05). The total effective rates of the treated and control groups were 80.36% and 74.14%, respectively. No significant difference existed between the two groups in “≥10%,” “≥0 and <10%,” and “<0” for hemorrhage area evaluation after treatment (*P* > 0.05).

### 3.6. Changes in Fluorescent Leakage Area

After treatment, fluorescent leakage area had notable narrowing in each group (*P* < 0.05), but no significant differences were found between both groups (*P* > 0.05) (Table 6 and Figure 2). The total effective rates of the treated and control groups in narrowing fluorescent leakage area were 69.64% and 70.69%, respectively. No significant difference was observed between the two groups in “≥10%,” “≥0 and <10%,” and “<0” for fluorescent leakage evaluation after treatment (*P* > 0.05).

### 3.7. Changes in Capillary Nonperfusion Area

Table 7 shows that the capillary nonperfusion area had a noticeable improvement in each group after treatment for three months (*P* < 0.05), but no significant difference was found between groups (*P* > 0.05). The total effective rates of the treated and control groups in narrowing fluorescent leakage area were 67.86% and 68.97%, respectively. No significant difference was observed between two groups in “≥10%,” “≥0 and <10%,” and “<0” for capillary nonperfusion area evaluation after treatment (*P* > 0.05).

### 3.8. Safety Evaluation

After the treatment, we analyzed each abnormal result in the blood routine, liver and kidney function, fasting blood sugar, and urine sugar tests. However, these tests had nothing to do with CDDP. Each laboratory examination had no significant differences between the two groups or before and after treatment of each group (*P* > 0.05) (Table 8), indicating that CDDP had no effect on the liver and kidney function of the diabetics and did not influence blood sugar content. No significant adverse events related to CDDP were recorded during the study.

## 4. Discussion

DR occurrence reflects the diabetic metabolic disorder and the effect of endocrine and hematological systems on retina. Although the microvascular complications in the retina caused by diabetes have been widely studied, the mechanisms and factors are not yet fully understood. The pathology of PDR is more severe than that of NPDR [14], that is, the growth of friable and abnormal new blood vessels. Therefore, active treatment should be provided for NPDR at the early stage of DR to delay retinopathy progression, maintain visual acuity, and improve the quality of life. The basic pathological changes of NPDR, such as selective pericyte loss, basement membrane thickening, microangioma formation, hyperplasia endothelialitis, and neovascularization, have been confirmed [16]. However, the pathogenesis of DR has not been elucidated; the blood hyperviscosity of diabetes was widely proven in clinical and empirical studies, which may lead to retinal ischemia and speed up DR progression [17, 18].

In case of high blood viscosity, the erythrocytes display low deformability and high aggregation, which can both decrease the blood flow; therefore, microthrombus is prone to occur, which might block the capillary vessels and lead to hypoxia-ischemia in retina tissue [8]. Microaneurysm, as a marker of early DR [19], is formed by the compensatory enlargement and expansion of retinal vessel bed in the condition of hyperischemia of retina tissue. To date, a pathological base, which can cause diabetic retinopathy, is poor against free radical, which also plays a key role in diabetes pathogenesis and platelet dysfunction [20]. The retina tissue under normal condition has an integrated antioxidase system [21], whereas the retina tissue of diabetics has a disturbed system [22]. At the same time, the fibrinolytic function of the diabetics is affected. The activity of tissue-type plasminogen activator notably declined, whereas the activity of the plasminogen activator inhibitor clearly increased. Both are crucial substances in the fibrinolytic system. The dynamic balance between them prevents thrombogenesis and maintains normal blood circulation. When the diabetic retinopathy is severe, the fibrinolytic activity is low [23]. The destruction of fibrinolytic function of blood plasma is due to the damage of vascular endothelial cell and pericyte loss.

CDDP mainly consists of* S. miltiorrhiza* (Danshen, 丹参),* P. notoginseng* (Sanqi, 三七), and borneol (Bingpian, 冰片), which have been used to treat various diseases (especially cardiovascular diseases) in China for hundreds of years.* S. miltiorrhiza* produces antioxidative effects, such as reducing malondialdehyde content and increasing superoxide dismutase activities to protect the vascular endothelial cells [24]. These antioxidative effects improve microcirculation disturbance, thereby increasing the blood flow to remove blood stasis [25].* P. notoginseng* is traditionally used to promote blood flow and hemostasis.* P. notoginseng saponins* (PNSs) are the main active components of* P. notoginseng* that also have antidiabetic potential on glucose production and absorption [26]. Borneol is an aromatic refreshing Chinese medicine that is commonly used to guide other components to target tissues [27]. CDDP may improve blood flow of the local tissue to prevent thrombosis [28, 29] and protect vascular endothelial cell through its ability of free radical elimination [30].

However, after 3 months of CDDP and dobesilate treatments, no significant statistical differences were observed between the two groups in the present study. Compared with before treatment, the treated group with CDDP showed obvious improvement in BCVA with a total effective rate of 71.43%, as well as the scope of visual field defect with the total effective rate of 62.50%. Thus, CDDP could effectively improve the hypoxia-ischemic condition of retina tissue. Because the retina tissue is sensitive to hypoxia-ischemia, a certain defect in the visual field was observed. From the pathological changes in retinal tissue, we also found that microaneurysm and hemorrhage were absorbed obviously after CDDP treatment and capillary nonperfusion area also narrowed, thereby demonstrating the microcirculation improvement further.

Clinically, some cases use CDDP to treat ophthalmic diseases caused by blood stasis or obstructed blood circulations, such as central retinal artery occlusion, retinal vein obstruction, central serous chorioretinopathy, central exudative chorioretinitis, and optic atrophy [31]. Observations showed that CDDP has certain curative effect in treating early DR, and these observations were used for clinical application and generalization. However, many trials had poor design and small sample size.

In conclusion, by observing the clinical related evaluation indexes, this study proved that CDDP has obvious clinical curative effect on early DR, including the control of microaneurysm and hemorrhage and improved visual acuity and visual field. This effect is similar to that of calcium dobesilate. In future DR treatments, CDDP may function as the auxiliary drug. Because of the limitation of capital and sample size, supporting evidence on the mechanism of curative effect in the body was lacking. To achieve the application and popularization of CDDP in the future clinical treatment of early DR, we will improve the design of experiment and perform an in-depth experimental study.

## Figures and Tables

**Table 1:** Basic characteristics of study subjects.

Characteristic	The treated group	The control group
Age (years)	59.54 ± 7.46 (range: 43–67)	57.86 ± 10.03 (range: 45–64)
Gender, number (%)		
Male	18 (64.3%)	19 (65.5%)
Female	10 (35.7%)	10 (34.5%)
Mean duration (years)	14.52 ± 3.29 (range: 7–21)	15.74 ± 3.63 (range: 9–21)
Diagnosis, eye/number		
Mild NPDR	18	19
Moderate NPDR	22	25
Severe NPDR	16	14

**Table 2:** BCVA (Log⁡MAR) comparison of the two groups before and after treatment.

Group	*n* (eye)	Before treatment	After treatment	BCVA evaluation after treatment			
				Improvement ≥ 4 lines	2 to 3 lines	1 line or no changes	Decline ≥ 2 lines
Treated group	56	0.25 ± 0.19	0.14 ± 0.17	8 (14.29%)	32 (57.14%)	14 (25.00%)	2 (3.57%)
Control group	58	0.32 ± 0.21	0.17 ± 0.18	6 (10.34%)	30 (51.72%)	20 (34.48%)	2 (3.45%)

**Table 3:** MD (dB) comparison of the two groups before and after treatment.

Group	*n* (eye)	Before treatment	After treatment	MD evaluation after treatment		
				≥10%	≥0 and <10%	<0
Treated group	56	4.61 ± 3.54	3.76 ± 3.94	9 (16.07%)	26 (46.43%)	21 (37.5%)
Control group	58	5.08 ± 4.58	3.68 ± 2.65	5 (8.62%)	34 (58.62%)	19 (32.76%)

**Table 4:** Microaneurysm number comparison of the two groups before and after treatment.

Group	*n *(eye)	Before treatment	After treatment	Microaneurysm number evaluation after treatment		
				≥10%	≥0 and <10%	<0
Treated group	56	23.16 ± 18.16	21.14 ± 18.57	15 (26.93%)	27 (48.07%)	14 (25.0%)
Control group	58	23.79 ± 17.30	21.88 ± 15.49	14 (24.14%)	31 (53.45%)	13 (22.41%)

**Table 5:** Hemorrhage area (PD) comparison of the two groups before and after treatment.

Group	*n* (eye)	Before treatment	After treatment	Hemorrhage area evaluation after treatment		
				≥10%	≥0 and <10%	<0
Treated group	56	0.30 ± 0.54	0.14 ± 0.52	20 (35.71%)	25 (44.64%)	11 (19.64%)
Control group	58	0.29 ± 0.48	0.15 ± 0.46	18 (31.03%)	25 (43.10%)	15 (25.86%)

**Table 6:** Fluorescent leakage area (PD) comparison of the two groups before and after treatment.

Group	*n* (eye)	Before treatment	After treatment	Fluorescent leakage area evaluation after treatment		
				≥10%	≥0 and <10%	<0
Treated group	56	0.16 ± 0.16	0.13 ± 0.20	16 (28.57%)	23 (41.07%)	17 (30.36%)
Control group	58	0.17 ± 0.17	0.12 ± 0.20	17 (29.31%)	24 (41.38%)	17 (29.31%)

**Table 7:** Capillary nonperfusion area (PD) comparison of the two groups before and after treatment.

Group	*n *(eye)	Before treatment	After treatment	Capillary nonperfusion area evaluation after treatment		
				≥10%	≥0 and <10%	<0
Treated group	56	0.21 ± 0.22	0.15 ± 0.15	12 (21.43%)	26 (46.43%)	18 (32.14%)
Control group	58	0.22 ± 0.23	0.14 ± 0.17	15 (25.86%)	25 (43.10%)	18 (31.03%)

**Table 8:** Comparison of the results of the laboratory examinations: comparison of the two groups before and after the treatment.

	Treated group		Control group	
	Before treatment	After treatment	Before treatment	After treatment
Red blood cell (×10^12^/L)	4.55 ± 0.48	4.64 ± 0.44	4.52 ± 0.47	4.50 ± 0.45
White blood cell (×10^9^/L)	6.81 ± 1.96	6.56 ± 2.03	7.02 ± 2.33	6.33 ± 1.44
Neutrophils (%)	57.21 ± 10.26	56.49 ± 9.91	59.23 ± 9.85	59.37 ± 8.66
Lymphocyte (%)	30.39 ± 7.17	32.62 ± 9.31	30.22 ± 8.19	29.24 ± 6.69
Hemoglobin (g/L)	142.54 ± 13.48	146.50 ± 11.70	139.90 ± 16.37	137.71 ± 13.70
Platelet (×10^9^/L)	230.07 ± 73.65	222.77 ± 64.40	232.90 ± 61.51	228.74 ± 68.22
Alanine aminotransferase (U/L)	23.30 ± 15.40	20.50 ± 8.74	21.54 ± 8.85	25.36 ± 14.04
Aspartate aminotransferase (U/L)	22.89 ± 9.62	21.04 ± 6.35	21.93 ± 7.25	25.07 ± 11.84
Blood urea nitrogen (mmol/L)	5.89 ± 1.70	5.95 ± 1.54	6.04 ± 1.81	6.11 ± 1.59
Blood creatinine (*μ*mol/L)	83.04 ± 12.26	82.19 ± 12.04	87.45 ± 16.65	88.03 ± 14.72
Fasting blood sugar (mmol/L)	6.74 ± 2.85	6.32 ± 2.76	6.48 ± 3.94	6.01 ± 2.64
Urine sugar (mmol/L)	7.93 ± 17.06	7.72 ± 15.46	8.61 ± 16.48	5.99 ± 13.05

## References

[1] Simo R., Ballarini S., Cunha-Vaz J. (2015). Non-traditional systemic treatments for diabetic retinopathy: an evidence-based review. *Current Medicinal Chemistry*.

[2] Klein R., Klein B. E. K., Moss S. E. (1984). Visual impairment in diabetes. *Ophthalmology*.

[3] Nentwich M. M., Ulbig M. W. (2015). Diabetic retinopathy—ocular complications of diabetes mellitus. *World Journal of Diabetes*.

[4] Poliakova M. A., Gavrilova N. A. (2012). The current conception about the pathogenic mechanisms of the diabetic optical neuropathy development. *Patologicheskaia fiziologiia i èksperimental'naia terapiia*.

[5] Abu El-Asrar A. M. (2013). Evolving strategies in the management of diabetic retinopathy. *Middle East African Journal of Ophthalmology*.

[6] Sato T., Morita S.-I., Bando H., Sato S., Ikeda T., Emi K. (2013). Early vitreous hemorrhage after vitrectomy with preoperative intravitreal bevacizumab for proliferative diabetic retinopathy. *Middle East African Journal of Ophthalmology*.

[7] Yun S. H., Adelman R. A. (2015). Recent developments in laser treatment of diabetic retinopathy. *Middle East African Journal of Ophthalmology*.

[8] Dedania V. S., Bakri S. J. (2015). Novel pharmacotherapies in diabetic retinopathy. *Middle East African Journal of Ophthalmology*.

[9] Duan J. G., Jin M., Jie C. H., Ye H. J. (2011). Standard of TCM diagnosis and treatment of diabetic retinal lesions. *Pharmacogenomics*.

[10] Luo J., Xu H., Chen K. (2013). Systematic review of compound danshen dropping pill: a chinese patent medicine for acute myocardial infarction. *Evidence-Based Complementary and Alternative Medicine*.

[11] Lian F., Wu L., Tian J. (2015). The effectiveness and safety of a danshen-containing Chinese herbal medicine for diabetic retinopathy: a randomized, double-blind, placebo-controlled multicenter clinical trial. *Journal of Ethnopharmacology*.

[12] Garay R. P., Hannaert P., Chiavaroli C. (2005). Calcium dobesilate in the treatment of diabetic retinopathy. *Treatments in Endocrinology*.

[13] Zhang X., Liu W., Wu S., Jin J., Li W., Wang N. (2014). Calcium dobesilate for diabetic retinopathy: a systematic review and meta-analysis. *Science China Life Sciences*.

[14] Wilkinson C. P., Ferris F. L., Klein R. E. (2003). Proposed international clinical diabetic retinopathy and diabetic macular edema disease severity scales. *Ophthalmology*.

[15] Rasmussen M. L., Broe R., Frydkjaer-Olsen U. (2015). Microaneurysm count as a predictor of long-term progression in diabetic retinopathy in young patients with type 1 diabetes: the Danish Cohort of Pediatric Diabetes 1987 (DCPD1987). *Graefe's Archive for Clinical and Experimental Ophthalmology*.

[16] The Diabetes Control and Complications Trial Research Group (1993). The effect of intensive treatment of diabetes on the development and progression of long-term complications in insulin-dependent diabetes mellitus. *The New England Journal of Medicine*.

[17] Duan H., Huang J., Li W., Tang M. (2013). Protective effects of Fufang Xueshuantong on diabetic retinopathy in rats. *Evidence-Based Complementary and Alternative Medicine*.

[18] Lowe G. D., Ghafour I. M., Belch J. J. F., Forbes C. D., Foulds W. S., MacCuish A. C. (1986). Increased blood viscosity in diabetic proliferative retinopathy. *Diabetes Research*.

[19] Haritoglou C., Kernt M., Neubauer A. (2014). Microaneurysm formation rate as a predictive marker for progression to clinically significant macular edema in nonproliferative diabetic retinopathy. *Retina*.

[20] Bonnefont-Rousselot D. (2002). Glucose and reactive oxygen species. *Current Opinion in Clinical Nutrition and Metabolic Care*.

[21] El-Bab M. F., Zaki N. S., Mojaddidi M. A., AL-Barry M., El-Beshbishy H. A. (2013). Diabetic retinopathy is associated with oxidative stress and mitigation of gene expression of antioxidant enzymes. *International Journal of General Medicine*.

[22] Halim E. M., Mukhopadhyay A. K. (2006). Effect of *Ocimum sanctum* (Tulsi) and vitamin E on biochemical parameters and retinopathy in streptozotocin induced diabetic rats. *Indian Journal of Clinical Biochemistry*.

[23] Yamagishi S.-I., Matsui T., Ueda S.-I., Nakamura K., Imaizumi T. (2007). Advanced glycation end products (AGEs) and cardiovascular disease (CVD) in diabetes. *Cardiovascular & Hematological Agents in Medicinal Chemistry*.

[24] Cao H., Zhang L., Sun Z.-B., Cheng X.-H., Zhang Y., Zou H.-B. (2015). Salvia miltiorrhiza prevents deep vein thrombosis via antioxidative effects in endothelial cells. *Molecular Medicine Reports*.

[25] Hao E.-W., Deng J.-G., Du Z.-C. (2012). Experimental study on two-way application of traditional Chinese medicines capable of promoting blood circulation and removing blood stasis with neutral property in cold and hot blood stasis syndrome I. *Zhongguo Zhong Yao Za Zhi*.

[26] Uzayisenga R., Ayeka P. A., Wang Y. (2014). Anti-diabetic potential of Panax notoginseng saponins (PNS): a review. *Phytotherapy Research*.

[27] Zhang Q., Wu D., Wu J. (2015). Improved blood-brain barrier distribution: effect of borneol on the brain pharmacokinetics of kaempferol in rats by in vivo microdialysis sampling. *Journal of Ethnopharmacology*.

[28] Zou H. M., Zhang B., Xu X. C. (2015). Urinary metabolomic strategy to evaluate Compound Danshen Dripping Pills for myocardial ischaemia in rats. *Journal of Pharmaceutical and Biomedical Analysis*.

[29] Ma S. T., Dai G. L., Cheng X. G. (2014). Synergistic action of Compound Danshen Dripping Pill (CDDP) on Clopidogrel Bisulfate (CPG) counteracting platelet aggregation. *Zhong Yao Cai*.

[30] Zhang Y., Wang J., Guo L. L., Wu G. J. (2013). Huoxue anxin recipe alleviated peroxidation damage of acute myocardial infarction rats by regulating iNOS/eNOS imbalance: an experimental research. *Zhongguo Zhong Xi Yi Jie He Za Zhi*.

[31] Liang R. G., Li C. H. (1998). The application report compound danshen dripping pill in ophthalmology clinic. *Journal of Chinese Medicinal Materials*.

